# The mediating role of self-disturbances in the relationship between stress sensitivity and psychotic-like experiences among individuals prone to psychosis

**DOI:** 10.1192/j.eurpsy.2026.12138

**Published:** 2026-07-31

**Authors:** R. Pionke-Ubych, J. Piwińska, L. Gawęda

**Affiliations:** Institute of Psychology, Polish Academy of Sciences, Warsaw, Poland

## Abstract

**Introduction:**

Previous studies have shown that self-disturbances mediate the relationship between traumatic life events and psychotic-like experiences. However, it is unknown whether this model may be extended to stress sensitivity. We hypothesize that a history of early trauma increases the risk for psychotic experiences through stress sensitization and disturbance of the basic sense of self.

**Objectives:**

The aim of the study was to investigate relationships between traumatic life events, stress sensitivity, self-disturbances and psychotic-like experiences.

**Methods:**

A sample of 50 (26 females; mean age: 27.64, SD = 4.42) individuals from the general population (n=1340, psychotic and neurological disorders as well as substance dependence were excluded) with the highest score in Prodromal Questionnaire (PQ-16) fulfilled questionnaires assessing stress sensitivity (Perceived Stress Scale; PSS-10), traumatic life events (Traumatic Experience Checklist; TEC) and self-disturbances (Inventory of Psychotic-like Anomalous Self-Experiences; IPASE). Pearson’s correlational analyses were performed to evaluate relationships between measured variables. Further mediation analysis with SPSS Process macro was performed.

**Results:**

All the variables significantly correlated with each other, except for the relationship between trauma and stress sensitivity, which turned out to be insignificant. Therefore, trauma was not included in the mediation model. Self-disturbances significantly mediated the relationship between stress sensitivity and psychotic-like experiences. The standardized total effect of stress sensitivity on psychotic-like experiences significantly differed from zero (β = 0.49, 95% CI 0.032 to 0.954, p<0.05). The standardized indirect effect of self-disturbances was significant (β = 0.51, 95% CI 0.145 to 0.991). The direct effect of stress sensitivity on psychotic-like experiences was insignificant (β = −0.01, 95% CI −0.370 to 0.341, p>0.05), which means that the mediation is indirect-only.

**Conclusions:**

The results of other studies indicating that a history of childhood trauma exposure is associated with subsequent stress sensitization were not reflected in our study data. However, elevated stress sensitivity was found to be indirectly related to psychotic-like experiences through enhanced self-disturbances.

**Disclosure of Interest:**

None Declared

